# Ultratrace Determination of Cr(VI) and Pb(II) by Microsample Injection System Flame Atomic Spectroscopy in Drinking Water and Treated and Untreated Industrial Effluents

**DOI:** 10.1155/2013/629495

**Published:** 2013-09-18

**Authors:** Jameel Ahmed Baig, Tasneem Gul Kazi, Latif Elci, Hassan Imran Afridi, Muhammad Irfan Khan, Hafiz Muhammad Naseer

**Affiliations:** ^1^National Centre of Excellence in Analytical Chemistry, University of Sindh, 76080 Jamshoro, Pakistan; ^2^Chemistry Department, Pamukkale University, 20017 Denizli, Turkey; ^3^Environmental Sciences, International Islamic University, 44000 Islamabad, Pakistan

## Abstract

Simple and robust analytical procedures were developed for hexavalent chromium (Cr(VI)) and lead (Pb(II)) by dispersive liquid-liquid microextraction (DLLME) using microsample injection system coupled with flame atomic absorption spectrophotometry (MIS-FAAS). For the current study, ammonium pyrrolidine dithiocarbamate (APDC), carbon tetrachloride, and ethanol were used as chelating agent, extraction solvent, and disperser solvent, respectively. The effective variables of developed method have been optimized and studied in detail. The limit of detection of Cr(VI) and Pb(II) were 0.037 and 0.054 *µ*g/L, respectively. The enrichment factors in both cases were 400 with 40 mL of initial volumes. The relative standard deviations (RSDs, *n* = 6) were <4%. The applicability and the accuracy of DLLME were estimated by the analysis of Cr(VI) and Pb(II) in industrial effluent wastewater by standard addition method (recoveries >96%). The proposed method was successfully applied to the determination of Cr(VI) and Pb(II) at ultratrace levels in natural drinking water and industrial effluents wastewater of Denizli. Moreover, the proposed method was compared with the literature reported method.

## 1. Introduction

Lead pollution is one of the most serious environmental problems because of their stability at contaminated sites, the reduction of enzymatic activities, and several other complications in human, plants, and animals [1]. It is introduced into water bodies (lakes, streams, and rivers) through the combustion of fossil fuels, smelting of sulfide ore, and acid mine drainage [2]. Characteristic indications of lead toxicity are abdominal pain, anaemia, headaches and convulsions, chronic nephritis of the kidney, brain damage, and central nervous system disorders [3]. The U.S. Environmental Protection Agency (EPA) has classified lead as a Group B2 human carcinogen [3]. Chromium occurs in higher concentration in the wastes from electroplating, paints, dyes, chrome tanning, and paper industries [4]. Cr(III) is an essential component having an important role in the glucose, lipid, and protein metabolism, whereas Cr(VI) is believed to be toxic and carcinogenic [5]. In addition, there is an increasing interest in the determination of Cr(VI) and Pb at trace level, because of persistence in the environment and bio-accumulative effect in living organisms [6].

Flame atomic absorption spectrometry (FAAS) is one of the most conventional techniques for the determination of trace metal ions because of the relatively simple and inexpensive equipment [7–9]. However, a preconcentration and/or separation step prior the final measurement is usually required for significant precise, sensitive, and accurate determination, in order to bring the analyte concentration into the dynamic range of the detector or to isolate the analyte from the desirable matrix constituents [3, 4, 10, 11].

The preconcentration methods like solvent extraction, ion exchange, adsorption, and coprecipitation were frequently used for trace level analysis of Cr(VI) and Pb(II) [12–17]. Liquid-liquid extraction, based on transfer of analyte from the aqueous sample to a water-immiscible solvent, is being widely employed for sample preparation [18]. But these methods are time consuming, needed significant chemical additives, large secondary wastes along procedure, and required complex equipment. Miniaturization of liquid extraction methods can be achieved by a drastic reduction of the extractant phase volume; three new methodologies have been developed, that is, single-drop microextraction, hollow fibre liquid-phase microextraction, and dispersive liquid-liquid microextraction [19]. Among them, dispersive liquid-liquid microextraction (DLLME) is effectively useful for the separation and pre-concentration of organic and inorganic contaminants in environmental samples in a single step [18, 20, 21].

For the current study, a new approach has been designed for the simultaneous and selective determination of Cr(VI) and Pb(II) at trace level in environmental samples based on DLLME. Ammonium pyrrolidine dithiocarbamate (APDC) has been used as a selective chelating agent for Cr(VI) and Pb(II) [22, 23]. The applicability of DLLME coupled with microsample injection system flame atomic absorption spectroscopy for Cr(VI) and Pb(II) preconcentration and separation was adopted as evaluated in our previous work [24]. The influence of the different analytical parameters for both methods was investigated in detailed. The proposed method was applied to tap water, groundwater, and industrial effluent before and after treatment for evaluation of Cr(VI) and Pb(II).

## 2. Experimental

### 2.1. Sampling

Six samples of each type of drinking water (tapwater and groundwater samples) were collected in November 2011 from different sampling sites of Denizli, Turkey (situated between the coordinates 37°46′48′′N and 29°04′48′′E in Aegean region). Whereas, the industrial effluent before and after treatment (six samples of each) from well organized industrial zone of Denizli with the assistance of Denizli Environment Quality Laboratory (DENCEV). The proposed methods (details are given below) were performed on the same week of sampling. So, the sample storage was not required.

### 2.2. Reagents

All solutions were prepared using ultrapure water (resistivity 18.2 MΩ/cm) obtained with a reverse osmosis system (Human Corporation, Seoul, Republic of Korea). Standard solutions of Cr(VI) and Pb(II) were prepared by dilution of 1000 ppm certified standard solutions, Fluka (Buchs, Switzerland). Working standard solutions were freshly prepared by appropriate dilution of the stock standard solution. Ammonium pyrrolidine dithiocarbamate (APDC, Fluka) was used as the chelating agent to form the hydrophobic metal complexes. A 5% (w/v) of APDC solution was prepared by dissolving suitable amount of APDC in ultrapure water. Buffer of pH 1-2 was prepared by adding an appropriate amount of hydrochloric acid, Merck (Darmstadt, Germany) and potassium chloride and buffer of pH 3–6 was prepared by adding an appropriate amount of acetic acid (Merck) and sodium acetate.

### 2.3. Apparatus

An atomic absorption spectrometer (PerkinElmer, AAnalyst 200), equipped with a chromium hollow cathode lamp, an air-acetylene flame atomizer, and handmade microinjection system, was used for determinations. The wavelength, lamp current, and spectral bandwidth used were 357.9 nm, 25 mA, and 0.7 nm, respectively. The pH measurements were carried out with a pH meter (WTW-pH-meter-720). A centrifuge (Hettich Centrifuge, Germany) was used to accelerate the phase separation process. Microinjector (Hamilton, Switzerland) was used for sediment phase separation.

### 2.4. Preparation of Handmade Microsample Injection System (MIS)

In routine practice, a sample volume of 2–4 mL is required for a single element determination by FAAS. However, a small volume was obtained by each preconcentration method (<0.5 mL), whereas there is need for high dilution. To resolve this problem, our group have made a microsample injection system (MIS), coupled to the nebulizer needle using a PTFE capillary tube (length of 12 cm) attached with yellow micropipette tip (capacity 20–200 *μ*L) as reported in our previous work [24]. For simultaneous determination of Cr(VI) and Pb(II), 100 mL was used for each analysis.

### 2.5. Dispersive Liquid-Liquid Microextraction

The Cr(VI) and Pb(II) were determined by DLLME, using a complexing reagent (APDC) and resulted complex was enriched into extraction solvent through disperser solvent. To optimize DLLME, four replicate of sub-samples (40 mL) of standard solution containing 10 *μ*g L^−1^ Cr(VI) and Pb(II) in PTFE tubes (50 mL in capacity). The pH was adjusted with appropriate buffer solution in the range of 1–6. Then, added 0.05–2% (w/v) APDC and 1–4 mL ethanol containing 100–400 *μ*L tetrachloride carbon was rapidly injected into the aqueous solution with a 2.5/5.0 mL syringe. As a result, a turbid phase immediately was formed. The acquired solution was centrifuged at 3000 rpm for 5 min. The CCl_4_ as sediment phase was separated in the range of 80–90 *μ*L by microinjector into 2 mL glass vial. The residual phase was vaporized to dryness by electrical hot plate and dissolved in 0.1 mL of 0.1 mol/L HNO_3_. The final solution was determined by MIS-FAAS.

### 2.6. Determination of Cr(VI) and Pb(II) in Water and Industrial Effluents

40 mL of replicate four subsamples of tap water, groundwater, and industrial effluent were filtered with ordinary filter papers to removed unsalable matrices and taken in PTFE tubes (50 mL in capacity). Then, treated with developed DLLME as illustrated above for determination of Cr(VI) and Pb(II), respectively.

## 3. Results and Discussion

In order to attain higher enrichment factor and significant recovery percentages of Cr(VI) and Pb(II) by DLLME coupled with MIS-FAAS, the characteristic parameters were investigated in detail. Each experiment was repeated four times and the results were presented with mean ± standard deviation at 95% confidence intervals.

### 3.1. Influence of the Extraction Solvent Type and Volume

The extraction solvents with higher density than water were mostly used for DLLME procedure. Among different solvents, CHCl_3_ and CCl_4_ were selected for pre-concentration of the Cr(VI) and Pb(II) on the basis of their high density and vapour pressure as well as less water solubility (19). Sample solutions were tested using 1.0 mL of ethanol (disperser solvent), which contains 100 *μ*L of understudied extraction solvents and rapidly injected into 40.0 mL of the aqueous sample resulted from complex formation reaction. In this experiment, CCl_4_ and CHCl_3_ resulted in enrichment factors of 400 ± 5.0 and 380 ± 8.0, respectively. CCl_4_ established the maximum enrichment factor as compared to CHCl_3_ because of its low solubility, less viscosity, and polarity (19). The formed sediment phase can easily be separated by a micro-injector. As a result, CCl_4_ was chosen as best extraction solvent.

**Figure 1:**
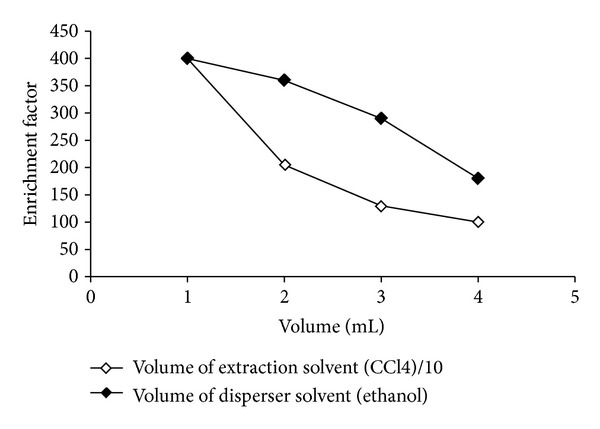
Influence of volume of the extraction solvent (CCl_4_) and dispersive solvent (ethanol) on the enrichment factor of Cr(VI) and Pb(II) at 40 mL initial volume of water samples 0.25% APDC, pH 3.00, and 0.01 mg/L Cr(VI)/Pb(II).

To evaluate the effect of the extraction solvent volume, solutions containing different volumes of CCl_4_ (100 to 400 *μ*L) were studied (Figure 1). It was observed that the enrichment factor of simultaneous preconcentration of Cr(VI) and Pb(II) by DLLME was decreased with increasing the volume of CCl_4_ (Figure 1). This is due to the large volume of sediment phase (80 to 320 *μ*L). At the end of experimental studies 100 *μ*L volume of CCl_4_ was selected for the subsequent investigations.

### 3.2. Influence of the Disperser Solvent Type and Volume

In DLLME, the dispersive solvent must be miscible both aqueous phase and extraction solvent. Thus, different solvents (CH_3_CHO, CH_3_CN, CH_3_CH_2_OH, and CH_3_OH) were tested as disperser solvent using 1.00 mL of each disperser solvent containing 100 *μ*L CCl_4_ (extraction solvent). However, the enrichment factors obtained by each studied dispersive solvent have no significant difference (*P* > 0.05). But, for the lowest toxicity and standard deviation of CH_3_CH_2_OH, further studies it have been chosen. The effect of the volume of CH_3_CH_2_OH on the extraction recovery was also carried out. The effect of different volumes of CH_3_CH_2_OH (1.00–4.00 mL) was carried out at optimum experimental conditions as shown in Figure 1. The enrichment factor decreased, when the CH_3_CH_2_OH volume increased. Thus, 1.00 mL of CH_3_CH_2_OH was chosen for further study.

### 3.3. Effect of the Extraction Time

Extraction time can be defined as the time interval between injecting the mixture of disperser solvent (CH_3_CH_2_OH) and extraction solvent (CCl_4_) into sample before starting to centrifuge [19]. Dependence of the enrichment factor upon extraction time was studied within a range of 5.0 sec–5.0 min, while the experimental conditions were kept constant. It is observed that extraction time has no significant effect on the enrichment factor and extraction efficiency of DLLME and phase separation is completed in 5.0 to 10.0 sec.

### 3.4. Salt Effect

The solubility of understudied analytes and organic extraction solvent in aqueous phase are usually decreased with the increase of salt concentration, which is favorable for reaching high recovery. Therefore, influence of salt concentration was evaluated at 0.05–4.00% (w/v) NaCl levels while other parameters were kept constant. The results showed that salt addition up to 1.00% did not affect the enrichment factor considerably. By increasing the ionic strength (from 1.00% to 4.00%), the solubility of the extraction solvent in the aqueous phase diminishes. As a result, the volume of the sediment phase increases from almost 110 to 130 *μ*L, which decreases the enrichment factor. The developed method can be applicable for separation of chromium from saline solutions up to 1.00%.

### 3.5. Influence of Sample pH and Concentration of APDC

**Figure 2:**
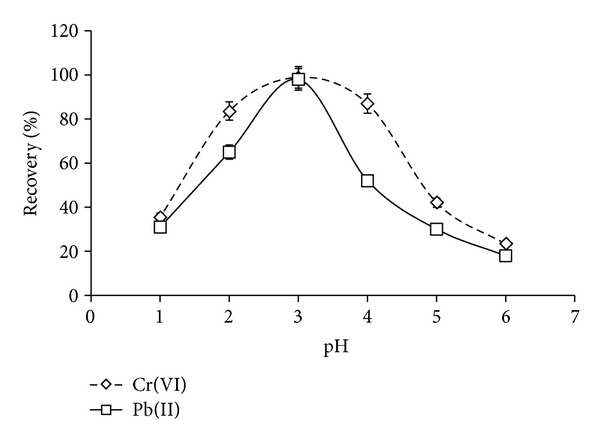
Influence of sample pH on DLLME at 40 mL initial volume of water samples 0.25% APDC, 100 *μ*L CCl_4_, 1.00 mL of ethanol, and 0.01 mg/L Cr(VI)/Pb(II).

The pH plays an important role in the extraction of inorganic compounds especially chromium in environmental samples for the complex formation reaction with complexing reagent including APDC. In this experiment, the effect of pH on the extraction performance within the range of 1–6 was investigated for Cr(VI) and Pb(II) as shown in Figure 2. It can be observed that the recovery of Cr(VI) was found to be high with the increase of pH value (2 to 4) and to abruptly fall down when pH was increased to 4 (Figure 2). At more acidic conditions, HCr_2_O_7_^−^ and Cr_2_O_7_^2−^ dimers become the dominant Cr(VI) form and the pK_a1_ and pK_a2_ values of these ions are 0.74 and 6.49, respectively [24, 25]. In aqueous solutions having a lower pH, the dichromate will be present primarily in its protonated form, that is, HCr_2_O_7_^−^, and it was observed in most of studies that the APDC mostly forms complexes with HCr_2_O_7_^−^ ions [24, 25]. In case of Pb(II), the obtained results show that the recoveries percentage of Pb(II) is high in the pH 3.0 (Figure 2). Therefore, the pH 3.0 was selected for simultaneous optimum recoveries of Cr(VI) and Pb(II).

**Figure 3:**
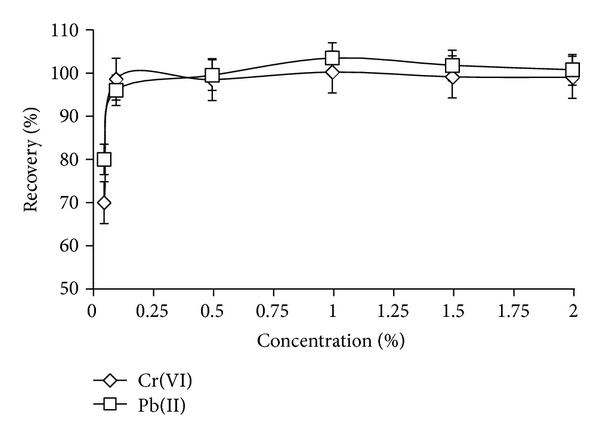
Influence of APDC concentration on DLLME for Cr(VI) and Pb(II) at 40 mL initial volume of water samples pH 2.0 and 0.10 mg/L Cr(VI)/Pb(II).

The effect of the APDC amount in the range of 0.05–2% (v/v) for the simultaneous preconcentration of Cr(VI) and Pb(II) was studied. In this case, the enrichment factor increased up to 0.1% (v/v) APDC, reaching a plateau, which is considered as maximum extraction (Figure 3). For simultaneously determination of Cr(VI) and Pb(II) was recorded in the range of 0.10–2.00% (m/v). On the behalf of resulted data, 0.25% (m/v) APDC solution in water was adopted for further study. The 0.25% (v/v) APDC was chosen as the optimal amount for the Cr(VI) and Pb(II) to prevent any interference.

### 3.6. Effect of the Coexisting Ions

In order to assess the feasibility of DLLME for analytical applications, the effect of some foreign ions which interfere with Cr(VI) and Pb(II) ion and/or often accompany analyte ions in various real environmental samples was investigated with the optimized conditions (Table 1). It is a general rule for analytical chemistry that any matrix was considered to interfere if the resulted FAAS signal of understudied analyte was varied ±5.0%. The recoveries of Cr(VI) and Pb(II) were almost quantitative in the presence of an excessive amount of the possible interfering cations and anions (Table 1).

### 3.7. Analytical Figure of Merits

The analytical characteristics of the proposed procedure were calculated under the optimized conditions. These characteristics were calculated using the values of the signals for analytical curve. The calibration and standard addition graphs were obtained for Cr(VI) and Pb(II), determined by microsample injection system flame atomic absorption spectrometry (MIS-FAAS). The linear range of the calibration graphs obtained in the concentration range from 1 to 10 *μ*g/L for Cr(VI) and Pb(II). The limit of detection (LOD) and limit of quantification (LOQ) were calculated as 3  ×  *S*_*b*_/*m* and 10  ×  *S*_*b*_/*m*, respectively, where *S*_*b*_ is the standard deviation of the blank (*n* = 10) and *m* is slope of the linear section of calibration graph. The LODs of Cr(VI) and Pb(II) were found to be 0.037 and 0.054 *μ*g/L, whereas, LOQs were 0.124 and 0.182 *μ*g L^−1^, respectively. The calibration curve of Cr(VI) and Pb(II) for over this interval was determined to be *A* = 2.10 × 10^−2^  × [Cr(VI)] + 6.00 × 10^−5^ and *A* = 2.40 × 10^−2^  × [Pb(II)] + 1.00 × 10^−3^, respectively, where *A* is the analytical signal, measured as absorbance, and [Cr(VI)] and [Pb(II)] are the concentrations of chromium and lead, respectively (*μ*g/L). The correlation coefficients (*r*) were 0.999 and 0.998 for Cr(VI) and Pb(II), respectively. The %RSD of both methods was evaluated with 40.00 mL from the solution containing the 0.01 *μ*g/L Cr(VI)/Pb(II) and was achieved <5% (*n* = 4). The enrichment factor (EF) was calculated by the ratio of Cr(VI)/Pb(II) concentration in sediment phase and initial concentration in original solution. Finally, the enrichment factor 400 was obtained for simultaneous preconcentration of Cr(VI) and Pb(II).

The reliability of the simplest calibration against aqueous standard solutions for DLLME was verified by means of spiking experiments at three concentration levels of Cr(VI) and Pb(II) in the range of 10 to 50 *μ*g/L with the maximum recovery percentage (98.5–110%) as summarized in Table 2. A good agreement was obtained between the added and measured analyte concentrations (*P* < 0.001).

### 3.8. Comparison of the Proposed Methods with the Other Literature Reported Method

Comparison of the proposed method with other approaches reported in the literature for preconcentration and determination of Cr(VI) and Pb(II) is represented in Table 3. The results showed that the extraction time for proposed DLLME is short (5–10 sec). The enrichment factors, LODs and %RSDs of DLLME, are comparable with those reported in the literature [22, 25–37]. The proposed methods (DLLME and CECP) coupled with MIS-FAAS are simple, most sensitive, and selective. Therefore, these methods could successfully be applied to the monitoring of trace amounts of chromium species in environmental, biological, and soil samples.

### 3.9. Application to Real Samples

The simultaneous preconcentration of Cr(VI) and Pb(II) in tap water, groundwater, and industrial effluents before and after treatment was studied using DLLME (Table 4). Cr(VI) and Pb(II) contents in tapwater and groundwater varied in the ranges 0.0 to 3.2 and 0.0 to 6.0 and (8.0 to 13.0 and 31.0 to 34.0 *μ*g/L, respectively. The Cr(VI) and Pb(II) contents in tap water samples were fallen within the WHO maximum contaminant level 50 and 10 *μ*g/L, respectively. The Cr(VI) and Pb(II) contents were found higher in groundwater as compared to tap water, because the natural abundance of geochemicals containing Cr(VI) and Pb(II). The high contents of Cr(VI) and Pb(II) in groundwater may cause hematological damage, brain damage, anemia, kidney malfunctioning, infections of skin and gastrointestinal tract, and carcinoma of the lung [38, 39]. But Cr(VI) in ground water of Denizli did not exceed WHO regulated values (50.0 *μ*g/L) of Cr(VI) for drinking water. Thus, both understudied waters (tapwater and groundwater) have safe levels of Cr(VI) for local community as natural drinking water sources. But Pb(II) in groundwater was found to be high, which is not suitable for drinking as well as cooking.

The Cr(VI) and Pb(II) in the tannery effluent samples before and after treatment were also determined by developed preconcentration method as shown in Table 4. The Cr(VI) and Pb(II) were observed in excess amount in untreated industrial water (Table 4), because of human activities especially through the production of wastewater in electroplating, tanning, textile, and dyestuff industries present in industrial zone. However, the Denizli municipality has a well-organized biological treatment plant based upon system of classical activated sludge for the recycling of industrial and municipality wastewater. The industrial samples after treatment have less amount of Cr(VI) and Pb(II); removal percentage of Cr(VI) and Pb(II) by biological treatment plant was 30 to 50% as shown in Table 4.

## 4. Conclusion

The DLLME coupled with MIS-FAAS was developed for simultaneous ultratrace level of Cr(VI) and Pb(II) determination in drinking water and industrial effluent before and after treatment. Proposed method proved to be fast, simple, inexpensive, and reproducible for the ultratrace level of Cr(VI) and Pb(II) with high enrichment factor (400). In this work, the use of handmade microsample injection systems for ultratrace level determination of Cr(VI) and Pb(II) offers several advantages including low cost, reusable and required less volume of sample with high recoveries percentage as compared to continuous flow FAAS.

## Figures and Tables

**Table 1:** Tolerable levels of concomitant ions for the extraction of 10 *µ*g/L Cr(VI) and Pb(II) (*N* = 4).

Ions	Tolerable levels (mg/L)	
	Cr(VI)	Pb(II)
Na^+^, Cl^−^	1,000	
Li^+^, K^+^, Ca^2+^, Mg^2+^	250	
SO_4_^−2^, NO_3_^−^	100	
Cu(II), Fe(II), Se(II), As(III), Co(II), Hg(II), Mn(II), Cr(III), Ni(II), Al(III), Zn(II)	50	

**Table 2:** The results for tests of addition/recovery for Cr(VI) by DLLME in industrial wastewater samples (*n* = 6).

	Amount added (*µ*g/L)	Found values Mean ± SD (*µ*g/L)	%Recovery^a^	%RSD
Cr(VI)	—	12.5 ± 2.4	—	—
	10	24.7 ± 0.3	110.0 ± 1.5	1.4
	25	40.7 ± 0.7	108.0 ± 1.8	1.7
	50	61.4 ± 3.4	98.5 ± 3.3	3.4

Pb(II)	—	10.4 ± 1.5	—	—
	10	20.5 ± 0.4	100.5 ± 2.2	2.0
	25	35.2 ± 0.8	99.4 ± 1.7	2.3
	50	59.8 ± 1.8	99.0 ± 1.2	3.0

^a^%Recovery = (*C*_after spiked_/(*C*_intial_ + *C*_spiked_)) × 100.

**Table 3:** Comparative data of analytical characteristics of the literature reported methods and proposed method.

Analyte(s)	Analytical technique	Extraction phase	Extraction time (min)	Enrichment factor	LOD (*µ*g/L)	RSD (%)	Ref.
Cr(VI)	FAAS	Ethyl xanthate complex onto naphthalene	15	100	0.500	3.1	[25]
Cr(VI)	CE-FAAS	Dy_2_O_3_ aqueous solution	20	100	0.780	—	[26]
Cr(VI)	UPAILDLLME-ETAAS	APDC/[Hmim][PF_6_]	5-6	300	0.07	9.2	[22]
Cr(VI)	DLLME-UV-Vis spectrophotometry	DIC/Toluene	—	—	30.0	<5.0	[27]
Cr(VI)	DLLME-ETAAS	APDC/CCl_4_	—	171	0.00596	3.02	[28]
Cr(VI) Cr(T)	DLLME-FAAS	APDC + EDTA/ CCl_4_	0.08	275 262	0.07 0.08	2.0 2.6	[29]
Cr(VI)	DLLME-ETAAS	APDC + ([C_8_MIm][NTf_2_])	—	300	0.002		[30]
Cr(VI)	DLLME-MIS-FAAS	APDC/CCl_4_	0.08–0.16	400	0.037	<4.0	Current study

Pb(II)	CP-FAAS	5-Chloro-2-hydroxyaniline-Cu(II)	10	50	2.72	<5.0	[31]
Pb(II)	MF-FAAS	Cellulose nitrate/HNO_3_	—	60	0.30	<10	[32]
Pb(II)	SDME-ETAAS	DDTP/CHCl_3_	7	52	0.20	<4.0	[33]
Pb(II)	IL-SDME-ETAAS	5-Br-PADAP/CYPHOS IL 101	15	32	0.0032	4.9	[34]
Pb(II)	IL-SDME-ETAAS	APDC/[C_4_MIM][PF_6_]	7	76	0.015	5.2	[35]
Pb(II)	DLLME-FAAS	DDTP/CCl_4_	0.08	450	0.50	3.8	[36]
Pb(II)	DLLME-GFAAS	DDTP/CCl_4_	<3	150	0.02	2.5	[37]
Pb(II)	DLLME-GFAAS	PMBP/CCl_4_	5	78	0.0039	3.2	[38]
Pb(II)	DLLME-MIS-FAAS	APDC/CCl_4_	0.08–0.16	400	0.054	<5.0	Current study

Ammonium pyrrolidine dithiocarbamate (APDC), 2-(5-bromo-2-pyridylazo)-5-diethylaminophenol (5-Br-PADAP), 1-butyl-3-methylimidazolium hexafluorophosphate [C_4_MIM][PF_6_], charge coupled device (CCD), coprecipitation flame atomic absorption spectroscopy (CE-FAAS), O,O-diethyldithiophosphate (DDTP), methylindocarbocyanine (DIC), 1,5-diphenylcarbazide (DPC), 1-hexyl-3-methylimidazolium hexafluorophosphate ([Hmim][PF_6_]), electrothermal atomic absorption spectroscopy (ETAAS), graphite furnace atomic absorption spectroscopy (GFAAS), inductively coupled plasma-optical emission spectrometry (ICP-OES), pyrrolidine dithiocarbamate (PDC), 1-phenyl-3-methyl-4-benzoyl-5-pyrazolone (PMBP), single drop microextraction (SDME), sequential injection analysis (SIA), tetradecyl(trihexyl)phosphonium chloride (CYPHOS IL 101), ultrasonic probe-assisted ionic liquid dispersive liquid-liquid microextraction (UPAILDLLME), 1-octyl-3-methylimidazolium bis(trifluoromethanesulfonyl)imide ([C_8_MIm][NTf_2_]).

**Table 4:** Analytical results of Cr(VI) and Pb(II) (*µ*g/L) in drinking water and industrial effluents (Denizli, Turkey).

Origin of water (no. of samples)	Cr(VI)	Pb(II)
Groundwater (*n* = 6)	3.7 ± 1.8	32.6 ± 1.6
Tap water (*n* = 6)	2.6 ± 0.5	10.4 ± 2.1
Untreated industrial effluent (*n* = 4)	23.3 ± 5.3	91.7 ± 1.8
Treated industrial effluent (*n* = 4)	9.6 ± 4.5	70.3 ± 1.2
